# Synergy of Fiber Surface Chemistry and Flow: Multi-Phase Transcrystallization in Fiber-Reinforced Thermoplastics

**DOI:** 10.3390/polym14224850

**Published:** 2022-11-10

**Authors:** Stan F. S. P. Looijmans, Michelle M. A. Spanjaards, Ljiljana Puskar, Dario Cavallo, Patrick D. Anderson, Lambèrt C. A. van Breemen

**Affiliations:** 1 Processing and Performance of Materials, Eindhoven University of Technology, P.O. Box 513, 5600 MB Eindhoven, The Netherlands; s.f.s.p.looijmans@tue.nl (S.F.S.P.L.); m.m.a.spanjaards@tue.nl (M.M.A.S.); p.d.anderson@tue.nl (P.D.A.); 2 Dutch Polymer Institute (DPI), P.O. Box 902, 5600 AX Eindhoven, The Netherlands; 3 Helmholtz-Zentrum für Materialien und Energie GmbH, Albert-Einstein-Straße 15, D-12489 Berlin, Germany; ljiljana.puskar@helmholtz-berlin.de; 4 Department of Chemistry and Industrial Chemistry, University of Genova, Via Dodecaneso 31, 16146 Genova, Italy; dario.cavallo@unige.it

**Keywords:** structure and morphology, polymer crystallization, polymer composites, shear-induced nucleation, transcrystallization, fiber–matrix interphase, polypropylene

## Abstract

Fiber-reinforced polymer composites are largely employed for their improved strength with respect to unfilled matrices. Considering semi-crystalline materials under relevant processing conditions, the applied pressure and flow induce shear stresses at the fiber–polymer interface. These stresses may strongly enhance the nucleation ability of the fiber surface with respect to the quiescent case. It is thus possible to assume that the fiber features are no longer of importance and that crystallization is dominated by the effect of flow. However, by making use of an advanced experimental technique, i.e., polarization-modulated synchrotron infrared microspectroscopy (PM-SIRMS), we are able to show that the opposite is true for the industrially relevant case of isotactic polypropylene (iPP). With PM-SIRMS, the local chain orientation is measured with micron-size spatial resolution. This orientation can be related to the polymer nucleation density along the fiber surface. For various combinations of an iPP matrix and fiber, the degree of orientation in the cylindrical layer that develops during flow correlates well with the differences in nucleation density found in quiescent conditions. This result shows that the morphological development during processing of polymer composites is not solely determined by the flow field, nor by the nucleating ability of the fiber surface alone, but rather by a synergistic combination of the two. In addition, using finite element modeling, it is demonstrated that, under the experimentally applied flow conditions, the interphase structure formation is mostly dominated by the rheological characteristics of the material rather than perturbations in experimental conditions, such as shear rate, layer thickness, and temperature. This once again highlights the importance of matrix–filler interplay during flow and, thus, of material selection in the design of hybrid and lightweight composite technologies.

## 1. Introduction

Fiber-reinforced polymer composites (FRCs) are widely employed due to their high specific stiffness and strength. With the growing demand for sustainable materials, the attention of both the scientific community and the petrochemical industry is drawn towards FRCs composed of a recyclable, thermoplastic matrix, e.g., isotactic polypropylene [1,2,3,4] or biodegradable alternatives, such as polylactic acid [5,6]. The semi-crystalline microstructure of these matrices makes the mechanical performance of FRCs strongly dependent on the processing conditions [7]. Despite the extensive literature on the crystallization of iPP/fiber composites, a detailed understanding of fiber-induced nucleation is still lacking. Both fiber surface chemistry [8,9,10] and topography, i.e., roughness [5,11,12,13], are thought to be important, but the exact roles of the two parameters are far from being elucidated [14]. In addition, the heterogeneous nucleation process is believed to be controlled by the number of chain ends and, thus, the molecular weight of the material considered [15]. Under relevant processing conditions, the application of pressure and flow induces shear stresses at the fiber–polymer interface, which strongly enhance the nucleation ability of the fiber surface with respect to quiescent conditions [16]. These stresses, in turn, give rise to a special crystal morphology, the so-called cylindritic morphology, which is often referred to as a transcrystalline layer (TCL). The subtle difference between these conventions was explained in detail by Varga et al. [17], but in this work, the latter is adopted for the structure that forms in both quiescent and shear flow conditions. Moreover, the mechanical performance of the fiber-reinforced polymer material is governed by the bonding between this TCL and the fiber [1,2,18,19]. In order to improve the adhesion between the two components, typically, the fiber surface and/or the matrix component are functionalized [8,9,10,20,21,22]. Hence, a comprehensive understanding of the effects of these compatibilization techniques on the developed morphology is indispensable for the design of long-, short-, and continuous-fiber composites.

The quantification of orientation around such interfaces is, however, rather limited when using optical microscopy techniques. While in quiescent conditions, the determination of the nucleation ability of the surface is well established [11,23,24,25], the vast number of nuclei that develop in shear flow conditions make the exact determination of structure practically impossible. As a result, the controversial data on the effect of interphase morphology on the mechanical properties of polymer composites may originate from the often ill-defined structure of the TCL. To fully characterize the microstructure that is probed when performing a mechanical test, quantitative data on the degree of chain orientation in the transcrystalline morphology with micrometer spatial resolution are essential. A wide variety of characterization techniques aiming to quantify this orientation have been developed over the years, e.g., birefringence measurements [26], vibrational linear dichroism (infrared and Raman based) [27,28,29,30], and X-ray scattering and diffraction [7], of which the latter can be considered the most mature. However, recently, Zhao et al. showed that linear dichroism measured with Fourier-transform infrared (FTIR) spectroscopy can quantitatively capture the decrease in orientation with increasing crystallization temperature when considering a TCL around a carbon fiber in an iPP melt [31]. At the same time, polarization-modulated synchrotron infrared microspectroscopy (PM-SIRMS) has emerged as a powerful tool for providing precise spatial information on variations in polymer chain orientation and, hence, the alignment of crystal axes in lamellar structures [32]. Contrary to conventional optical techniques, with PM-SIRMS, it is possible to quantitatively distinguish highly oriented transcrystalline morphologies. In the recent past, the technique has been successfully applied to study transcrystallinity in iPP/fiber composites [29,33,34].

In the present work, this versatile technique is adopted to study transcrystallization in iPP matrices of different molecular weights around both neat and surface-modified glass fibers, as well as aramid fibers. The obtained measure from the PM-SIRMS resembling the fiber surface nucleation ability corresponds well with the nucleation density that is deduced from quiescent crystallization studies of different fibers. Hence, the morphological development during processing of these systems still depends to a large extent on the chemical and topological compatibility of the two phases and is not solely determined by the shear flow. In order to verify that small variations in experimental conditions do not contribute to the significant differences measured in the nucleation density at the fiber–matrix interphase, numerical simulations were performed. Using a finite element method (FEM) framework, the effect of a deviation in fiber velocity, fiber displacement, shear time, and sample height on the wall shear stress (responsible for molecular alignment and, thus, TCL formation) was investigated. Within reasonable limits, an inconsistency in either of these aforementioned parameters cannot cause the wall shear stress to vary more than a factor of two, i.e., it does not explain the significant differences in the degree of lamellar orientation measured using PM-SIRMS.

## 2. Materials and Methods

### 2.1. Materials and Sample Preparation

In analogy with many other studies, isotactic polypropylene (iPP) was chosen as a model system, since the material is relevant from both an industrial and an academic perspective. Its rheological properties and crystallization kinetics have been well characterized by many authors over the last few decades. Two iPP grades with different molecular weights, i.e., 130 kg/mol (iPP-1) and 365 kg/mol (iPP-2), which have a comparable polydispersity index of 5.5, were kindly provided by Borealis Polyolefine GmbH (Linz, Austria). A third maleic anhydrided (MAH)-modified matrix from Exxonmobil (Irving, TX, USA) with a low viscosity was examined and compared to the composite and injection moulding grades mentioned before. To create the single-fiber composites, a neat glass fiber (NGF) and a surface-modified glass fiber (SGF) that were functionalized for polyolefin impregnation and produced by Nippon Electric Glass (Otsu, Japan) are employed. In addition, a neat aramid fiber (NAF) and epoxy-functionalized aramid fiber (SAF) from Teijin Aramids (Arnhem, The Netherlands) were used in order to compare the influences of fiber chemistry.

**Figure 1:**
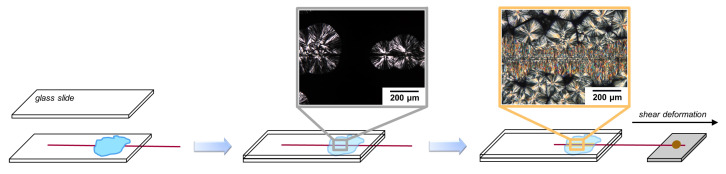
Sample preparation procedure for thin polymer films containing a single fiber. By controlling the fiber velocity and temperature history, a well-defined cylindrical microstructure was created.

Samples for optical microscopy and polarization-modulated infrared spectroscopy experiments were prepared through manual compression molding on a conventional hot plate. A small volume was cut from the polymer granulate and melted on a microscope glass slide at a temperature of 200 °C. A second preheated glass slide was placed on top, and the polymer was compressed into a thin film with a thickness of approximately 50 μm. The sample was then cooled to room temperature, and the thin film was removed from the substrate. Subsequently, a glass slide was clamped onto the heating element of a Linkam LT350 hot stage on which a single filament of the above-mentioned fibers was placed. The fiber length in both the experiments and simulations is considered “infinitely” long, i.e., much longer than one would ever observe in a practical fiber-reinforced application. However, the aim of this study is to characterize the morphology development around a fiber and to unravel the underlying mechanisms that contributed to that particular cylindritical morphology. In this case, the obtained results are independent of the fiber length, and a longer fiber aids in performing easier sample preparation. The polymer film was melted on top at a temperature of 200 °C and kept isothermal for two minutes to erase its thermomechanical history and ensure complete fiber impregnation. As schematically indicated in Figure 1, the samples were isothermally crystallized at a temperature of 130 °C, either in quiescent or shear flow conditions. Flow conditions were examined by gluing one of the fiber ends to the internal micromanipulator of the LT350 stage, which could be manually rotated at a maximum speed of ≈ 2 RPM. Shear was applied by displacing the fiber over a distance of 1.5 mm at a rate of ≈ 1 mm/s, i.e., three full revolutions of the manipulator as soon as the sample reached a temperature of 130 °C, while keeping the glass substrate to which the molten polymer film adhered fixed. The single-fiber composite was then removed from the glass substrate using a razor blade such that it could be mounted while free-standing on the FTIR microscope, as described in the next section.

The quiescent nucleation rate was measured by means of polarized optical microscopy. Thin films of polymer were melted around a single fiber as described above. After keeping the sample at 200 °C for two minutes to erase any thermomechanical stresses resulting from the compression step, the sample was cooled down at a rate of 30 °C/min to the isothermal crystallization temperature (ranging between 126 and 141 °C, depending on the combination of the fiber and matrix). An Olympus BX51 microscope was used in transmission mode to illuminate the sample between cross-polarizers while collecting a time series of images. The nucleation rate was then determined by directly counting the number of nuclei appearing at the fiber surface, taking into account the outer surface area of the fiber. An analogue of this method was described previously [5,12].

### 2.2. Polarization Modulation Setup

Polarization-modulated synchrotron infrared microspectroscopy experiments were carried out at the IRIS THz/Infrared beamline, which is part of the Helmholtz Zentrum Berlin facility BESSY II [35]. The setup is a series coupling of an FTIR spectrometer (Nicolet Nexus 870, Thermo, Waltham, MA, USA), a photoelastic modulator (PEM-90 II, Hinds Instruments, Hillsboro, OR, USA), and an infrared microscope (Nicolet Continuum, Thermo, Waltham, MA, USA), equipped with two 32× Schwartzchild objectives and a liquid-nitrogen-cooled HgCdTe detector, which was used in transmission mode. In the rest of this section, we will discuss solely the photoelastic modulator (PEM); other details on the beamline setup can be found elsewhere [32,35].

The PEM was actuated by piezoelectric transducers, stretching and compressing the ZnSe crystal in an oscillatory fashion. The birefringence that is created at an operating frequency of 50 kHz and peak retardation of λ/2 leads to a change between two perpendicular linear polarization states at a frequency of 100 kHz. An electronic head was connected to a controller that regulated the peak retardation and transmitted the signal to a demodulator (SSD 100, GWC Technologies, Madison, WI, USA). The signal recorded by the microscope detector was fed into the demodulator, and using the retardation set by the controller, the signal was demodulated into two interferograms, one representing the differential polarization signal of the two linearly polarized states, and the second being the sum interferogram that was used for normalization. As both signals are recorded simultaneously, this technique becomes insensitive to environmental changes and thickness variations within the sample.

Interferograms were collected in the spectral range of 4000–650 cm ${}^{-1}$ , with each spectrum being the average of 128 scans. The use of synchrotron radiation makes it possible to go down to a 10 μm aperture size, which approaches the diffraction limit of the incident light [35]. The peak retardation of the PEM was set at a wavenumber of 1200 cm ${}^{-1}$ . As the measurement was performed continuously, it should be noted that the effective birefringence is at its maximum only at this specific frequency, and that spectral bands far away from the set-point lacks complete optical correction. In each sample, the fiber direction was aligned to one of the optical axes of the setup, which, to a good approximation, coincided with the horizontal axis of the stage.

### 2.3. Method Validation

The use of polarization-modulated infrared spectroscopy has only recently been applied in the field of polymer science [33,34]. In conventional measurements of linear dichroism, where the sample is manually rotated with respect to the polarization of the light, errors may arise from various sources, of which lateral inaccuracies due to difficult sample positioning and continuously changing composition of the environment over time are the most pronounced. In addition, this method requires each location on the sample to be exposed to the light at least twice. Using PM-SIRMS, most of these challenges disappear; since the polarization of the light is altered at a high frequency, i.e., much higher than the mirror velocity of the interferometer, both the parallel and perpendicular spectra are recorded simultaneously. In this way, the sample needs only a single measurement per location, saving time, and no artificial rotation of the polarization is needed, preventing lateral inaccuracies. This technique directly provides a so-called differential absorption spectrum, which is defined as:

(1) $$I_{\mathrm{PMDD}}=\frac{I_{\|}-I_{⊥}}{I_{\|}+I_{⊥}},$$

where I_‖_ is the intensity at the detector when the incident light is oriented parallel to the optical axis of the sample, and I_⊥_ is the intensity at the detector when the light is polarized perpendicularly with respect to the sample.

**Figure 2:**
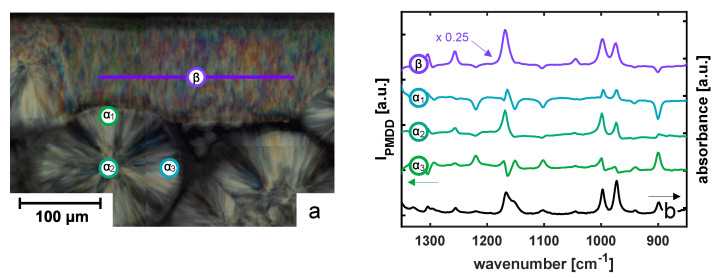
(**a**) Polarized optical micrograph of a representative morphology close the fiber–matrix interphase. (**b**) PMDD spectra taken at different positions in the semi-crystalline morphology, showing several spectral bands that are sensitive to vibrational linear dichroism. The locations where the PMDD spectra were measured are indicated by the markers in (**a**). A conventional FTIR absorbance spectrum of iPP is provided as a reference.

To validate the technique, reference points were measured at a representative morphology of iPP-2 with an embedded aramid fiber, as shown in Figure 2. Differential absorption spectra taken at the locations indicated in Figure 2a are shown in Figure 2b, where the bottom curve is a conventional FTIR absorption spectrum. As can be seen from Figure 2b, several bands of the differential absorption spectrum are prone to linear dichroism, i.e., show a different absorption depending on the polarization of the light. When considering, for instance, the bending of the methyl group, corresponding to the peak at 900 cm ${}^{-1}$ , the differential absorption is positive when the α -lamellae are vertically oriented, i.e., spectrum α _1_, while it almost vanishes when the location of the sample is quasi-isotropic, i.e., spectrum α _2_, and is negative when the lamellae are oriented horizontally, i.e., spectrum α _3_ in Figure 2b.

To see the difference in lamellar orientation in the TCL, a line scan in the transcrystalline β-phase layer was performed. Due to the larger birefringence and dichroism of the β-phase lamellae caused by the difference in crystalline lattice and morphology, the resulting differential absorption was significantly higher; see Figure 2b. It should be noted that for comparison reasons, the curve corresponding to the β-phase-phase was scaled by a factor of 0.25 in absolute value. Furthermore, since the β-phase lamellae are known to be twisting over relatively large length scales, we will present values of the differential absorption averaged over a length of 200 μm in the remainder of this work in order to have a representative statistical average.

### 2.4. Numerical Methods

**Figure 3:**
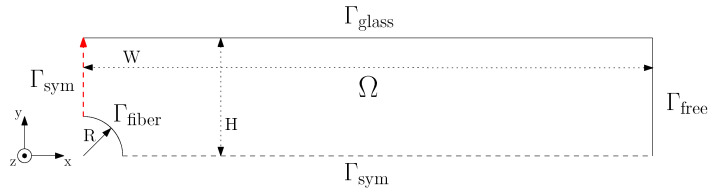
Schematic representation of the fiber-pulling problem. Only a quarter of the actual geometry is modeled because of internal symmetry.

**Figure 4:**
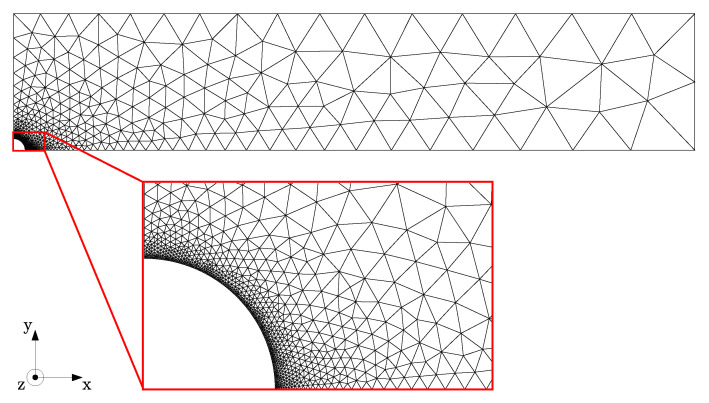
Mesh used in the numerical simulations of the fiber-pulling problem. As mentioned in the description of the methods, a 3D problem was solved on a 2D mesh, which heavily reduced the number of elements and, hence, allowed for considerable mesh refinement close to the fiber surface, as shown in the zoom.

Finite element simulations were performed to investigate the influence of experimental errors on the flow profile and resulting stress field around the fiber in order to exclude these as a source of inconsistencies in the measured flow-induced nucleation density. The problem was solved as a 3D velocity, pressure, and conformation field, yet on a 2D mesh; under the assumption that the length of the fiber is large compared to the diameter, the fiber is assumed to be infinitely long, and thus all gradients in the fiber direction can be considered irrelevant, i.e., equal to zero. This allowed for relatively short computation times—between one and four days—while still having a sufficiently refined mesh close to the fiber surface. The problem is schematically shown in Figure 3, where the domain Ω is described by the fiber radius R, the half sample height H, and the half sample width W. Subsequently, the domain was spatially discretized into triangular P2–P1 (Taylor Hood) elements [36], with a gradient in mesh size from coarse at the glass substrate and free lateral surface to fine at the fiber interface, i.e., the location where the spatial gradients were highest; see Figure 4. The geometry was meshed using the Gmsh software package [37]. Mesh convergence was assumed at the point where the change in the wall shear rate was less than 1%.

On the top boundary, the domain Ω, which resembles the glass substrate, the velocity in all directions is set equal to zero. At the lateral free surface, a zero-traction boundary condition is applied, while at the left and bottom sides of the domain, a fully symmetric flow condition is prescribed. The movement of the fiber is controlled by imposing a velocity in the z-direction. The boundary conditions are given by:

(2a) $$\begin{array}{cccc} \vec{u}\; & =\;\vec{0}\; & & \mathrm{on}\;\Gamma_{\mathrm{glass}}, \end{array}$$

(2b) $$\begin{array}{cccc} \vec{t}\; & =\;\vec{0}\; & & \mathrm{on}\;\Gamma_{\mathrm{free}}, \end{array}$$

(2c) $$\begin{array}{cccc} \vec{u}\; & =\;{\vec{u}}_{\mathrm{fiber}}\; & & \mathrm{on}\;\Gamma_{\mathrm{fiber}}, \end{array}$$

where the traction vector $\vec{t}$ on a surface with normal $\vec{n}$ is defined as $\vec{t}=\sigma \cdot \vec{n}$ .

The fluid is considered to be incompressible and body forces are neglected. In addition, because of the low Reynolds number O(−6) , we neglect the convective term from the Navier–Stokes equation. Based on that same argument, the transient term is neglected as well, yet it is kept in the equation because of the high-velocity gradient that is introduced when a step–strain-rate problem is simulated. Taking this into consideration, the balance of mass and the balance of momentum are given by:

(3a) $$\begin{array}{cccc} \vec{\nabla }\cdot \vec{u}\; & =\;0\; & & \mathrm{in}\;\Omega , \end{array}$$

(3b) $$\begin{array}{cccc} \rho \frac{\partial \vec{u}}{\partial t}\; & =\;\vec{\nabla }\cdot \sigma \; & & \mathrm{in}\;\Omega , \end{array}$$

where $\vec{u}$ is the fluid velocity, *t* is the time, and σ is the Cauchy stress as given by:

(4) $$\sigma \;=\;-pI+2\eta_{s}D+\tau ,$$

where *p* is the pressure, I is the unity tensor, $\eta_{s}$ is the Newtonian viscosity, D is the rate-of-deformation tensor, and τ is the viscoelastic stress tensor which is described as an extra stress tensor based on the shear modulus *G* and the conformation tensor c according to:

(5) τ=G(c−I).

The evolution equation for the conformation is given by the Giesekus model [38,39]:

(6) $$\overset{▿}{c}+\frac{1}{\lambda }\left(c-I\right)+\frac{\alpha }{\lambda }{\left(c-I\right)}^{2}=0,$$

with λ being the relaxation time of the polymer and α being the nonlinear parameter used to take shear-thinning effects into account. The time step was chosen to be 10^3^ times smaller than the shortest relaxation time, i.e., 10 ${}^{-5}$ s, at which full time step convergence was reached.

## 3. Results and Discussion

**Figure 5:**
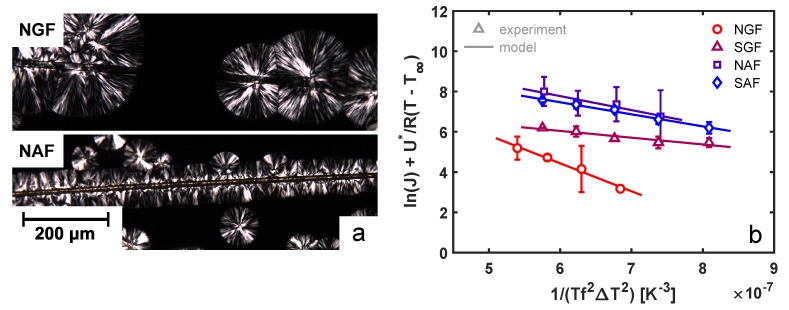
(**a**) Example of POM images showing the isothermally crystallizing microstructure at 130 °C around a neat glass fiber (NGF) in the top panel and a neat aramid fiber (NAF) in the bottom panel. (**b**) Quantification of the quiescent heterogeneous nucleation rate as a function of temperature for the four different fibers considered here. The lines are model fits that yield the rate parameters given in Table 1.

To study the effect of fiber surface chemistry on the nucleation ability towards an iPP melt, samples were isothermally crystallized at various temperatures under quiescent conditions. An example that highlights the difference in the nucleation ability of various substrates is presented in Figure 5a, where a glass fiber (top panel) is compared to an aramid fiber (bottom panel). Whereas the glass fiber shows no interaction with the matrix material surrounding it, the aramid fiber clearly shows an enhanced nucleation efficiency at the fiber surface. By capturing optical microscopy images between two cross-polarizers in time, the nucleation rate was quantified by simply counting the number density of nuclei in time. The obtained nucleation rates per unit surface area are shown in Figure 5b as a function of temperature for the various fibers. With the lowest surface roughness and no chemical compatibility with the matrix, the neat glass fiber shows the lowest nucleation ability, followed by the functionalized glass fiber and, subsequently, the aramid fibers. These local differences between the various fibers in terms of the bulk and interphase morphology may lead to substantial variations in mechanical performance. Due to the development of an oriented transcrystalline layer perpendicular to the fiber direction, the modulus and strength in the direction parallel to the fiber orientation may have been increased, while the ultimate tensile strain was drastically decreased.

To quantitatively describe these differences in fiber–matrix affinity, classical heterogeneous nucleation theory was used; the nucleation ability of a given substrate towards a polymer matrix is proven to be effectively captured by a parameter Δ σ that is directly proportional to the free-energy barrier for nucleation. The temperature-dependent nucleation rate is then given by [40]:

(7) $$J\left(T\right)\;=\;J_{0}\exp\left(-\frac{U^{*}}{R(T-T_{\infty })}\right)\exp\left(-\frac{16\sigma \sigma_{e}\Delta \sigma {T_{m}^{0}}^{2}}{kTf^{2}{\left(T_{m}^{0}-T\right)}^{2}{\Delta h_{0}}^{2}}\right),$$

where $J_{0}$ is a pre-factor that is independent of temperature, $U^{*}$ represents the energy barrier for diffusion of chain segments over the phase boundary, *R* is the universal gas constant, $T_{\infty }$ is a fictive temperature at which all viscous flow is assumed to cease, typically 30 °C below the glass-transition temperature, σ and $\sigma_{e}$ are the free energies of the lateral and fold surface, respectively, under the conditions that they are fully in contact with the undercooled amorphous melt, $T_{m}^{0}$ is the equilibrium melting temperature, *k* is Boltzmann’s constant, *f* is a correction factor ( $=2T_{c}/\left(T_{c}+T_{m}^{0}\right)$ ), and $\Delta h_{0}$ is the specific enthalpy of fusion for a perfect crystal at its equilibrium melting point.

The parameters for the equilibrium melting point $T_{m}^{0}=458$ K, the fictive temperature $T_{\infty }=232$ K, the energy barrier $U^{*}=6.28\times 10^{3}$ J mol ${}^{-1}$ , and the enthalpy of fusion $\Delta h_{0}=1.96\times 10^{8}$ J m ${}^{-3}$ were adopted from the classical works of Clark and Hoffman [41] and Cheng et al. [42]. The product of σ and $\sigma_{e}$ was deduced from the work of Wang et al. [43] to be 732 mJ^2^ m ${}^{-4}$ . Fitting Equation (7) to the experimental data presented in Figure 5b yields the parameters for the pre-factor $J_{0}$ and the energy barrier for nucleation Δ σ ; see Table 1. When considering the neat (NGF) and sized glass fiber (SGF), it is clear that the application of a surface coating effectively lowers the free-energy barrier for nucleation, resulting in a high nucleation density at the surface. However, comparing the aramid fibers to the glass fibers, an energy barrier that is between that of the NGF and SGF is found (based on the above-described model). This suggests that the chemical compatibility of both the NAF and SAF with the iPP matrix is worse than that of the SGF, yet the number of nuclei that are observed at the fiber surface is higher than in the case of the SGF for all temperatures assessed. In fact, the Δ σ values found for the aramid fibers are close to those reported for bulk heterogeneous nucleation of polypropylene, i.e., 1.23 mJ m ${}^{-2}$ [43]. It may be evident that the difference in pre-exponential factor $J_{0}$ is the determining figure here; due to the manufacturing process of the aramid fibers, a slight surface texture and heterogeneity were present, which may have enhanced the nucleation process [12]. Scanning electron microscopy (SEM) images of the different fibers are shown in the Supplementary Material. The Twaron fibers possess significantly greater amounts of heterogeneities at the surface in comparison with the findings of Wang et al. [25] on Kevlar 49 fibers. This difference is ascribed to the different manufacturing techniques used by the aramid producers and can explain the significant difference in the Δ σ values obtained when comparing our work to the literature.

**Figure 6:**
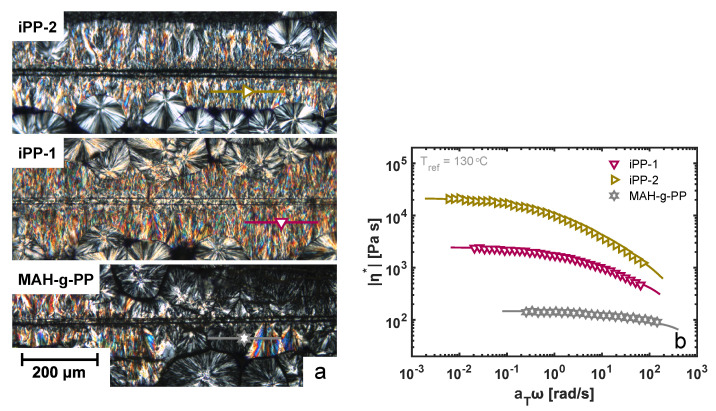
(**a**) Effect of matrix molecular weight on the developed was in a single-fiber composite sample. The horizontal lines indicate the location where a line scan is made to obtain a statistical average for the dichroic difference, i.e., the parameter used to quantify the degree of orientation of the cylindrical layer. (**b**) The complex viscosity as a function of frequency for the different matrix materials under consideration. The lines represent the fitted Maxwell modes that describe the relaxation of these undercooled melts. The model parameters are given in Table S1 of the Supplementary Material.

Under shear flow conditions, on the other hand, this strong influence of the fiber surface characteristics seemingly vanishes. The alignment of polymer chains close to the fiber surface leads to the formation of a so-called columnar morphology, as a vast amount of β -phase nuclei can form on highly oriented α -phase crystals. Visually, it becomes extremely difficult to observe structural differences in such highly oriented systems, as can be seen from the cross-polarized micrographs presented in Figure 6a. Whereas an intuitive distinction between the structures formed around a glass fiber for matrix materials of different molecular weights may be still possible, a quantitative measure of the nucleation density is rather unachievable. By means of polarization-modulated infrared spectroscopy, a technique increasingly applied in the field of polymer science [33,34], we were able to detect lamellar orientation in transcrystalline and columnar morphologies, as was recently demonstrated [29]. Combining this technique with a highly brilliant synchrotron light source, a spatial resolution down to the diffraction limit is achieved, and hence, the degree of orientation in the single fiber systems can be determined down to a length scale of approximately 10 × 10 μm^2^. Polarization-modulated synchrotron infrared microscopy (PM-SIRMS) line scans with a length of 200 μm were traced parallel to the fiber surface at a distance of 50 μm from the surface and a step size of 5 μm at the locations indicated in Figure 6a. The degree of alignment was calculated from the differential absorbance spectrum, as defined by Equation (1), at a wavelength of 1169 cm ${}^{-1}$ , which corresponds to the carbon–carbon symmetrical stretch vibrational band and may, thus, be considered a measure for the backbone stretch of the polypropylene main chain.

**Figure 7:**
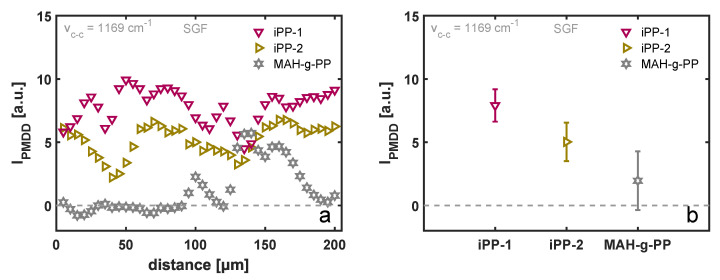
Intensity of the polarization-modulated dichroic difference as (**a**) a function of sample position corresponding to the lines indicated in Figure 6a and (**b**) averaged over a length of 200 μm with the error bars indicating the variation of the signal with position, as can be deduced from (**a**).

Three different molecular weights of the matrix material are considered in this work. The complex viscosity was measured as a function of frequency using small-amplitude oscillatory shear (SAOS), and the resulting master curves at a shifted temperature of 130 °C are presented in Figure 6b. The variation in zero-shear viscosity between the three materials is roughly a factor of 10, and hence, under comparable shear flow conditions, the flow field and resulting crystalline morphology is very different, as deduced from Figure 6a. The results of the PM-SIRMS line scans are presented in Figure 7a as a function of the scan distance. For the iPP-1 and iPP-2 samples, which showed the most homogeneous TCL morphology in POM, the polarization-modulated dichroic ratio is fairly constant, yet due to the small spot size of the IR beam, local differences are detected. These variations could arise from local differences in the arrangement of the lamellae, as well as the twisting of the β-phase lamellae [44]. For the MAH-g-PP sample, however, where due to the fast relaxation of the polymer chains after application of shear flow a distorted interphase layer was formed, that dichroic ratio is close to zero in the region where no TCL was observed and increases when the scan position reached the partially oriented β-phase crystals. This implies that the above-described characterization technique is capable of detecting these structural changes, although it is not particularly useful for the characterization of weakly oriented morphologies. The average PMDD ratio is presented in Figure 7b, where the markers correspond to the mean values measured over a distance of 200 μm, while the error bars indicate the standard deviation of the individual measurement points. Contrary to what may be expected, yet in line with earlier findings by Folkes and Hardwick [15], a substantially lower degree of chain alignment was measured for the material with the highest molecular weight, i.e., iPP-2, as compared to the lower-molecular-weight material, iPP-1. Typically, the high-molecular-weight fraction of a polymer material leads to a stronger alignment of chains, and thus, a greater orientation of the TCL can be anticipated. Taking into account the larger resistance of the iPP-2 material against flow, presented by the master curves of the complex viscosity in Figure 6b, it is likely that the smooth and stiff fiber slipped through the soft melt when being pulled rather than deforming the polymer chains at the interphase. This hypothesis is substantiated by the results of the FEM simulations, but this is outside the scope of this work and is not elaborated upon further.

**Figure 8:**
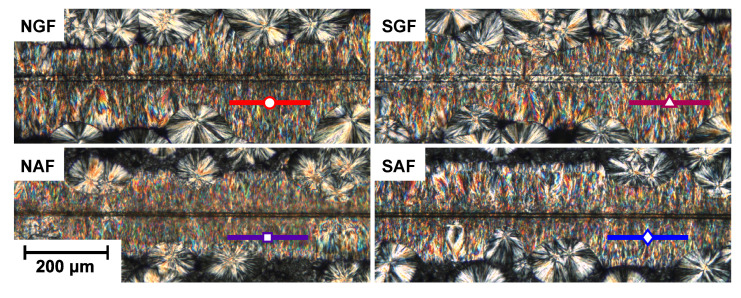
Cross-polarized micrographs of the various fibers embedded in an iPP-1 matrix after shearing for 1.5 mm at a rate of 1 mm/s. The lines indicate the positions scanned with the PM-SIRMS technique.

**Figure 9:**
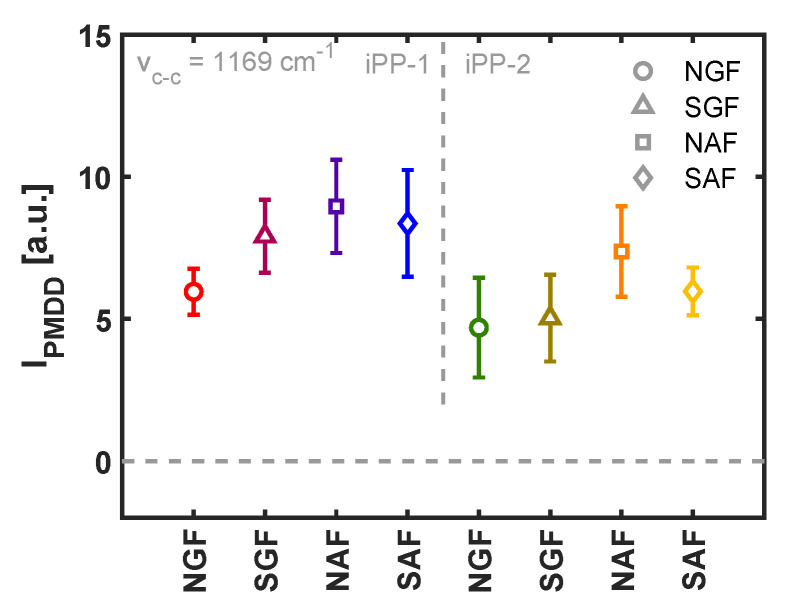
Differential dichroic intensity of the vibrational band corresponding to backbone stretching for various combinations of the two homopolymer materials and fiber types. Analogously to Figure 7, the markers represent the average degree of orientation, while the error bars indicate the positional spread of the data.

Polarized optical micrographs of the morphology around four different fibers sheared through an iPP-1 matrix are shown in Figure 8. It may be clear that for these systems, it is practically impossible to rank or quantify the degree of lamellar alignment based solely on such images, and that when these morphologies are observed, one jumps to the conclusion that adhesive parameters at the fiber–matrix interphase become irrelevant, and crystal structure formation is governed by the applied flow. Therefore, to verify whether that assumption was valid, for both iPP-1 and iPP-2, PM-SIRMS scans of 200 μm at a distance of 50 μm from the fiber interface were collected, the locations of which are indicated in Figure 8. The average peak intensity of the backbone stretching, i.e., the vibrational band at 1169 cm ${}^{-1}$ , is shown in Figure 9 for the different fibers. The horizontal dotted line represents a PMDD intensity of zero, indicating an isotropic microstructure at the length scale of the IR light. For both matrix materials, a similar trend was observed when comparing the fiber types; the neat glass fiber showed the lowest degree of lamellar alignment, followed by the sized glass fiber and aramid fibers, respectively. For the iPP-1 samples, the average PMDD intensity is higher for all fiber types compared to the iPP-2 (higher molecular weight) sample, which can be attributed to slippage between fiber and matrix during pulling, as discussed before. The degree of orientation measured in the TCL correlates well with the nucleation rate observed in quiescent conditions. As the nucleation density at the fiber surface increases, the steric hindrance of neighboring lamellae prevents a nucleus from growing in the lateral direction as a spherulite, and growth solely takes place from the fiber surface outward, creating a columnar morphology around the fiber. Hence, the vibration corresponding to the stretching of the backbone of the chain is directly related to the nucleation density at the fiber surface. This result demonstrates that the TCL structure formation upon shear flow is not solely determined by the flow field and flow strength, nor by the chemical interaction between fiber and matrix alone, but rather by a synergistic combination of the two.

During the sample preparation of these TCL morphologies, parameters such as sample thickness, shear rate, shear time, and isothermal crystallization temperature were kept constant. However, given the high sensitivity of the PM-SIRMS measurement and the resolution of the equipment used to prepare the morphology, small deviations in the flow field upon pulling may have emerged. In order to verify that the observed differences in crystal alignment resulted solely from a difference in fiber surface chemistry rather than a variation in the applied flow field due to experimental inconsistencies, FEM simulations of the fiber-pulling experiment were performed. The incompressible mass and momentum balance were solved on the domain shown in Figure 3 using a nonlinear viscoelastic (Giesekus) constitutive model [38,39]. The nonlinear parameter α represents the decrease in viscosity with increasing deformation rate and was fitted to the complex viscosity data under the assumption that for these linear polymer systems, the Cox–Merz rule [45] is applicable; for both iPP-1 and iPP-2, this parameter is set equal to 0.49. The deformation profile and viscoelastic stress state are evaluated for the two highest matrix molecular weights, i.e., iPP-1 and iPP-2, for varying sample thicknesses, pulling distances, -rates, and -times. Here, solely the results regarding an inconsistency in the maximum velocity and sample thickness for the iPP-1 sample are discussed, while the influence of time lag and displacement amplitude, as well as all results for the iPP-2 sample, can be found in the Supplementary Material.

**Figure 10:**
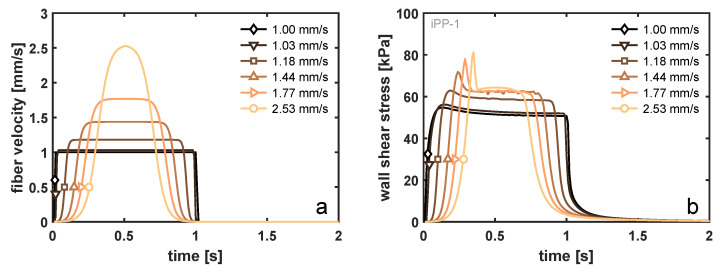
(**a**) The prescribed velocity profiles at the fiber surface and (**b**) the corresponding wall shear stress at the fiber surface as a function of time. These simulation results represent the iPP-1 matrix material.

The fiber-pulling experiment was set up such that the total displacement of the fiber and the time to reach that displacement are fixed. It is assumed that the mass of the fiber is sufficiently low that its acceleration is infinitely fast, and hence, a step velocity profile is applied to the fiber. The fact that, in reality, the acceleration of the fiber is finite may influence the imposed velocity profile in the vicinity of the interface, as well as the local stress state, due to the simultaneous stress build-up from the increase in deformation (rate) and stress decrease because of viscoelastic relaxation. In the simulations, the gradual increase in fiber velocity was modeled using an arctangent velocity profile, as illustrated in Figure 10a, while keeping the total displacement of the fiber equal to 1.00 mm and the total shear time equal to 1 s. As a result, the maximum velocity at the fiber wall increases, and that effect on the viscoelastic shear stress at the wall is examined and is shown in Figure 10a. Comparing the velocity profile with a maximum velocity of 2.53 mm/s with that of a step-velocity profile (1.00 mm/s), the lag in acceleration reduces the initial increase in wall shear stress due to a competition between stress build-up and relaxation. In fact, due to the relatively low relaxation times of the iPP-1 sample (Table S1 of the Supplementary Material), a decrease in wall shear stress was already observed prior to the fiber reaching its maximum velocity. Pronounced shear thinning of this material leads to a peak stress that is only 45% increased at a fiber velocity of 2.53 mm/s, as compared to the 1.00 mm/s reference (increase of 153% in velocity), and an increase of only 20% of the steady-state plateau stress. For the iPP-2 material, where shear thinning is present at already lower deformation rates, this increase in wall shear stress is even less pronounced. It should be noted that the lag in acceleration described here is highly exaggerated in relation to the possible experimental error, and that, even in this extreme case, a marginal increase in the stress was observed.

**Figure 11:**
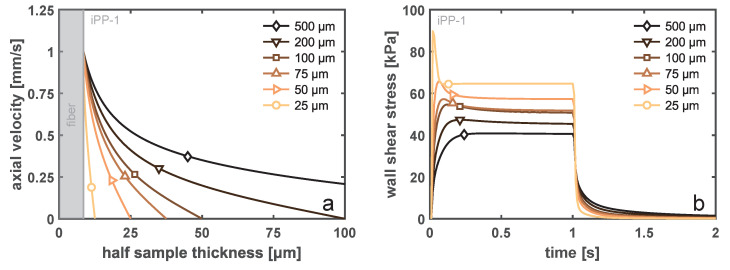
(**a**) The steady-state velocity profiles over the sample thickness during a step-shear-rate experiment, i.e., 1 mm/s for 1 s, for varying sample thicknesses, and (**b**) the corresponding time evolution of the wall shear stress at the fiber surface.

A similar conclusion may be drawn from the FEM results describing a step-velocity experiment at 1 mm/s for 1 s for varying sample thicknesses. Figure 11a displays the steady-state velocity profile over the sample height, where the sample width was always 500 μm and the fiber diameter was 17 μm. With decreasing sample thickness, the shear rate at the fiber surface increases in order to fulfill the imposed boundary conditions at both ends (no slip). This effect was historically modeled using the theory of Monasse [46], who took the effect of shear thinning into account for a viscous fluid. In the Supplementary Material, we present the velocity profiles around a fiber for iPP-1 and iPP-2. These two homopolymer grades have the same power-law exponent for the model of Monasse, which implies that the velocity profile should look identical. From the viscoelastic simulations, it becomes clear that this can not be true, and a more pronounced boundary layer starts to form when the resistance against flow increases, i.e., the viscosity increases and mobility becomes dominated by relaxation. The low molecular weight of the iPP-1 material leads to a fast relaxation and, hence, a nonlinear shear profile, even for thin samples. However, although the wall shear rates may be very different with changing sample thickness, due to the fact that the imposed deformation mostly occurs at rates that are in the shear thinning region of the material, the net increase in the wall shear stress is minute, as can be deduced from Figure 11b. When the thickness was reduced from 500 to 50 μm, i.e., a factor of 10, the wall shear stress increases solely by 50% . In the case in which the sample thickness was decreased to be approximately equal to the fiber diameter, confinement effects and fiber–wall interactions start to be significant, which increases the local stress substantially. Again, this is an extreme condition that was not encountered experimentally.

Taking all FEM simulation results into consideration, it becomes evident that variations in the molecular weight (distribution), i.e., the relaxation time spectrum of the material, contribute far more to the local shear stress at the fiber surface than any experimental variable that we impose. Hence, the substantial differences in crystal orientation measured by PM-SIRMS must have originated from the intrinsic interplay between the matrix and fiber and were only negligibly influenced by the imposed experimental conditions. The influence of temperature was outside of the scope of this numerical study, as the control of the Linkam heating stage was far more precise than the temperature dependence of the relaxation times, i.e., even with a (large) deviation of 1 °C in shear temperature, the difference in viscoelastic properties was negligible. In addition, the temperature was always checked using the growth rate of the spherulites, which was well established for iPP, as an “intrinsic thermocouple”.

These insights into structural development and characterization will aid in the quantitative understanding of the mechanical performance and provide processing guidelines for fiber-reinforced polymer (FRP) constructs. The single-fiber flow experiments resulted in a detailed understanding of the semi-crystalline morphology that developed at the matrix–fiber interface, be it a short, long, or continuous FRP. The combination of fiber length, orientation distribution, and interface structure determines the eventual mechanical properties of a multi-fiber product.

## 4. Conclusions

In this work, the nucleation ability of various fibrous substrates towards isotactic polypropylene was determined in both quiescent and shear flow conditions. A substantial difference in interphase morphology of iPP around a single fiber was found, which could be entirely attributed to the nucleation efficiency of the various fibers; a non-sized (neat) glass fiber presented a nucleation rate that is one to two orders of magnitude lower compared to that of an aramid fiber. As a result, a transcrystalline morphology was created around the aramid fibers, while in the case of a glass fiber, the surface nucleation density was similar to that of the bulk polymer material.

In shear flow, the alignment of α -phase crystals at the fiber interphase lead to a strong increase in nucleation efficiency for all investigated fiber types, and one may argue that the fiber surface characteristics are no longer important. By using polarization-modulated infrared microspectroscopy, it was shown that, upon processing of polymer composites, the developing morphology is not solely determined by the flow field, nor by the nucleating ability of the fiber surface alone, but rather by a synergistic combination of the two, as the influence of fiber–matrix affinity is retained even when a strong shear flow is applied.

These experimental findings were substantiated by FEM simulations, which proved that under the applied sample preparation conditions, the stress that leads to chain alignment and subsequent nucleus formation is almost completely governed by the rheological properties of the matrix. Minute inconsistencies in the sample thickness, pulling distance, and pulling velocity (profile) that were within experimental error seemed to only marginally influence the crystallizing morphology. Other parameters such as the matrix molecular weight, polydispersity and the resulting relaxation time spectrum, the presence of wall slip at the fiber–matrix interface, and the choice of fiber surface chemistry dominate the TCL formation and, hence, are key for controlling the structure and properties of fiber-reinforced thermoplastic materials. 

## Figures and Tables

**Table 1:** Nucleation density parameters.

Sample Name	*J*_0_ [μm ${}^{-2}$ s ${}^{-1}$ ]	Δσ [mJ m ${}^{-2}$ ]
NGF	3.453 × 10^5^	2.807
SGF	3.153 × 10^3^	0.679
NAF	1.539 × 10^5^	1.408
SAF	6.337 × 10^4^	1.212

## Data Availability

The data presented in this study are available on request from the corresponding author.

## Supplementary Materials

The Supplementary Material can be downloaded at: https://www.mdpi.com/2073-4360/14/22/4850/s1.
