# Supplementary material for: Synergy of Fiber Surface Chemistry and Flow: Multi-Phase Transcrystallization in Fiber-Reinforced Thermoplastics
Source: Polymers (Basel). 2022 Nov 10;14(22):4850. doi: 10.3390/polym14224850 (PMC9694137; doi:10.3390/polym14224850)
Supplement: Supplementary file 1 [file polymers-14-04850-s001.zip › polymers-2000196-supplementary.pdf]

Supplementary Material

Synergy of fiber surface chemistry and flow: multi-phase transcrystallization in fiber-reinforced thermoplastics

Stan F.S.P. Looijmans[a,b], Michelle M.A. Spanjaards[a], Ljiljana Puskar[c], Dario Cavallo[d], Patrick D. Anderson[a], Lambèrt C.A. van Breemen[a,*]

[a] *Processing and Performance of Materials, Eindhoven University of Technology, P.O. Box 513, 5600 MB Eindhoven, The Netherlands*
[b] *Dutch Polymer Institute (DPI), P.O. Box 902, 5600 AX Eindhoven, The Netherlands*
[c] *Helmholtz-Zentrum für Materialien und Energie GmbH, Albert-Einstein-Straße 15, D-12489 Berlin, Germany*
[d] *Department of Chemistry and Industrial Chemistry, University of Genova, Via Dodecaneso, 31 - 16146 Genova, Italy*

Abstract

Fiber-reinforced polymer composites are largely employed for their improved strength with respect to the unfilled matrix. Considering semi-crystalline materials under processing relevant conditions, the applied pressure and flow induce shear stresses at the fiber/polymer interface. These stresses may strongly enhance the nucleation ability of the fiber surface with respect to the quiescent case. It is thus possible to assume that the fiber features are no longer of importance, and that crystallization is dominated by the effect of flow. However, making use of an advanced experimental technique, i.e. polarization modulated synchrotron infrared microspectroscopy (PM-SIRMS), we are able to show that the opposite is true for the industrially relevant case of isotactic polypropylene (iPP). With PM-SIRMS, the local lamellar orientation is measured with micron-size spatial resolution. This orientation can be related to the polymer nucleation density along the fiber surface. For various combinations of iPP matrix and fiber, the degree of orientation in the cylindrical layer that develops during flow correlates well with the differences in nucleation density found in quiescent conditions. This result shows that the morphology development during processing of polymer composites is not solely determined by the flow field, nor by the nucleating ability of the fiber surface alone, but rather by a synergistic combination of the two. In addition, using finite element modelling it is demonstrated that, under the experimentally applied flow conditions, the interphase structure formation is mostly dominated by the rheological characteristics of the material rather than perturbations in experimental conditions such as shear rate, layer thickness and temperature. This once again highlights the importance of matrix-filler interplay during flow and thus material selection in the design of hybrid and lightweight composite technologies.

Keywords: Structure and morphology, Polymer crystallization, Polymer composites, Shear-induced nucleation, Transcrystallization, Fiber-matrix interphase, Polypropylene

*Corresponding author
Email address: L.C.A.v.Breemen@tue.nl (Lambèrt C.A. van Breemen)

Scanning Electron Microscopy

Scanning Electron Microscopy (SEM) images are taken at the fiber surface. In Figure S1a a general overview of the fiber surface characteristics is given, while a more detailed impression is given in Figure S1b.

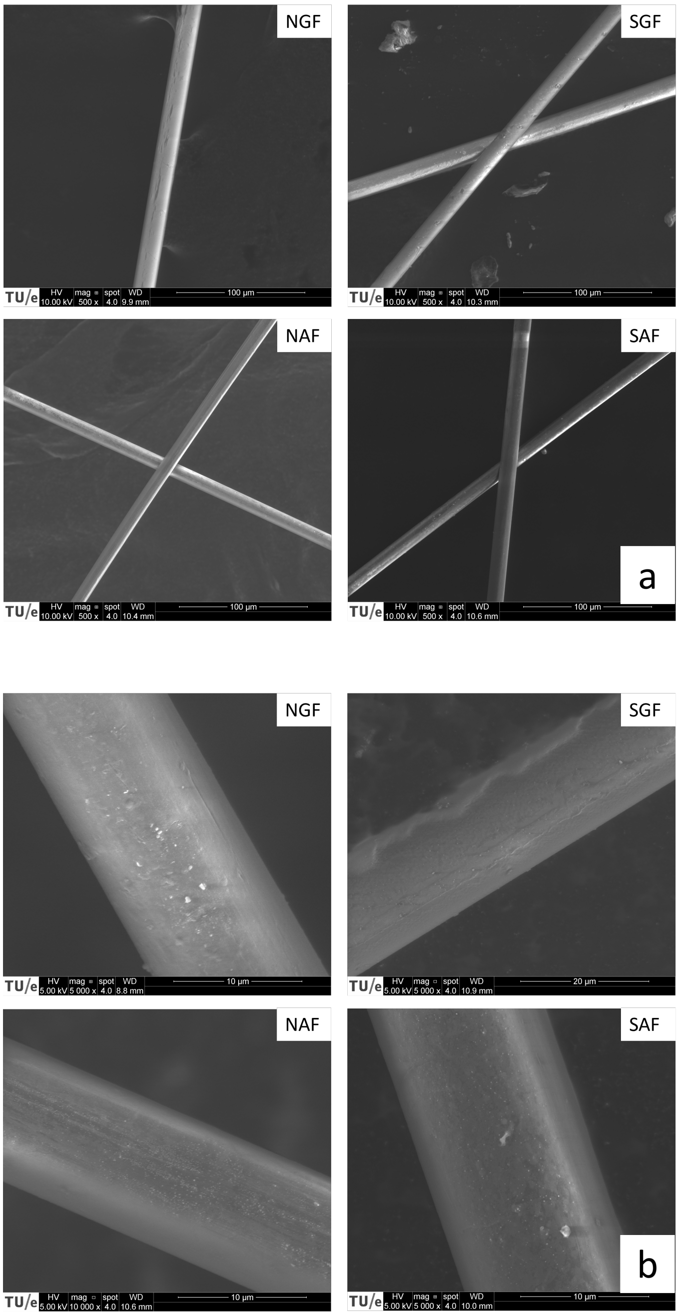

Figure S1: SEM images of the four different fiber surfaces under study at a magnification of a) 500x and b) 10.000x.

Determination of material model parameters

In this work three matrix polypropylenes are considered. iPP-1 with a weight average molecular weight of 130 kg/mol, iPP-2 with 365 kg/mol and a maleic anhydride grafted material with a significantly lower molecular weight than iPP-1 and iPP-2, as can be deduced from the viscosity plot in Figure 6b of the article. The relaxation time spectrum of the materials is discretized using a multi-mode Maxwell model and applying a least-squares fit to the experimental data obtained from SAOS, resulting in the model parameters presented in Table S1. It should be noted that the non-linear Giesekus parameter is chosen to be constant for all obtained Maxwell-modes.

Table S1: Maxwell parameters complex viscosity at a reference temperature of 130 °C

iPP-1 mode	λ_i [s]	G_i [Pa]
1	$8.542 \cdot 10^{-3}$	$5.190 \cdot 10^{4}$
2	$7.371 \cdot 10^{-2}$	$8.190 \cdot 10^{3}$
3	$3.949 \cdot 10^{-1}$	$1.560 \cdot 10^{3}$
4	$2.172 \cdot 10^{0}$	$2.100 \cdot 10^{2}$
5	$1.840 \cdot 10^{1}$	$1.760 \cdot 10^{1}$

iPP-2 mode	λ_i [s]	G_i [Pa]
1	$1.327 \cdot 10^{-2}$	$8.340 \cdot 10^{4}$
2	$9.519 \cdot 10^{-2}$	$2.880 \cdot 10^{4}$
3	$4.979 \cdot 10^{-1}$	$1.020 \cdot 10^{4}$
4	$2.509 \cdot 10^{0}$	$2.380 \cdot 10^{3}$
5	$1.333 \cdot 10^{1}$	$3.250 \cdot 10^{2}$
6	$1.152 \cdot 10^{2}$	$1.740 \cdot 10^{1}$

MAH-g-PP mode	λ_i [s]	G_i [Pa]
1	$2.167 \cdot 10^{-3}$	$3.770 \cdot 10^{4}$
2	$1.591 \cdot 10^{-2}$	$1.630 \cdot 10^{3}$
3	$7.127 \cdot 10^{-2}$	$2.850 \cdot 10^{2}$
4	$4.881 \cdot 10^{-1}$	$3.870 \cdot 10^{1}$

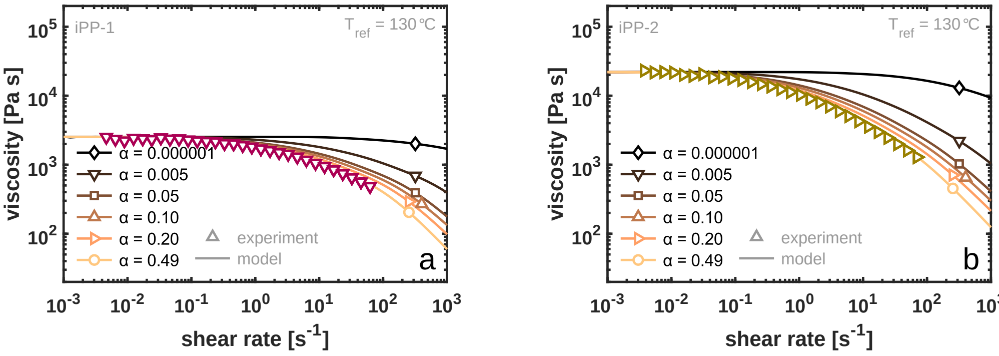

Figure S2: Fitting of the experimentally measured viscosity (SAOS) using the Giesekus constitutive model with different non-linear parameters for a) iPP-1 and b) iPP-2.

Effect of the non-linear Giesekus parameter α

Under the assumption of the Cox-Merz rule, the non-linear Giesekus parameter can be determined as shown in Figure S2. For both matrix materials under consideration, an α of 0.49 (as close to the physical limit of 0.50 as possible) is able to describe the shear thinning behavior well. The importance of this parameter on the velocity field around the fiber is shown in Figure S3a for iPP-1 and Figure S3c for iPP-2. The corresponding wall shear stress as a function of time is plotted in Figures S3b and Figure S3d respectively. By increasing the molecular weight, under the same applied flow the stress will be larger, and as a result the thinning sets in at lower strain rates. This leads to the formation of a boundary layer of high shear rate in the vicinity of the fiber substrate. This thinning, combined with the increased elasticity with increasing molecular weight, leads to a different stress (and strain) field around the fiber, which has a pronounced effect on the degree of molecular orientation one can reach at the surface. The above results demonstrate that the combination of zero-shear viscosity, i.e. molecular weight, and the amount of shear thinning in the material have a substantial effect on the wall shear stress and thus the nucleation efficiency of the substrate, as well as the possibility of slippage.

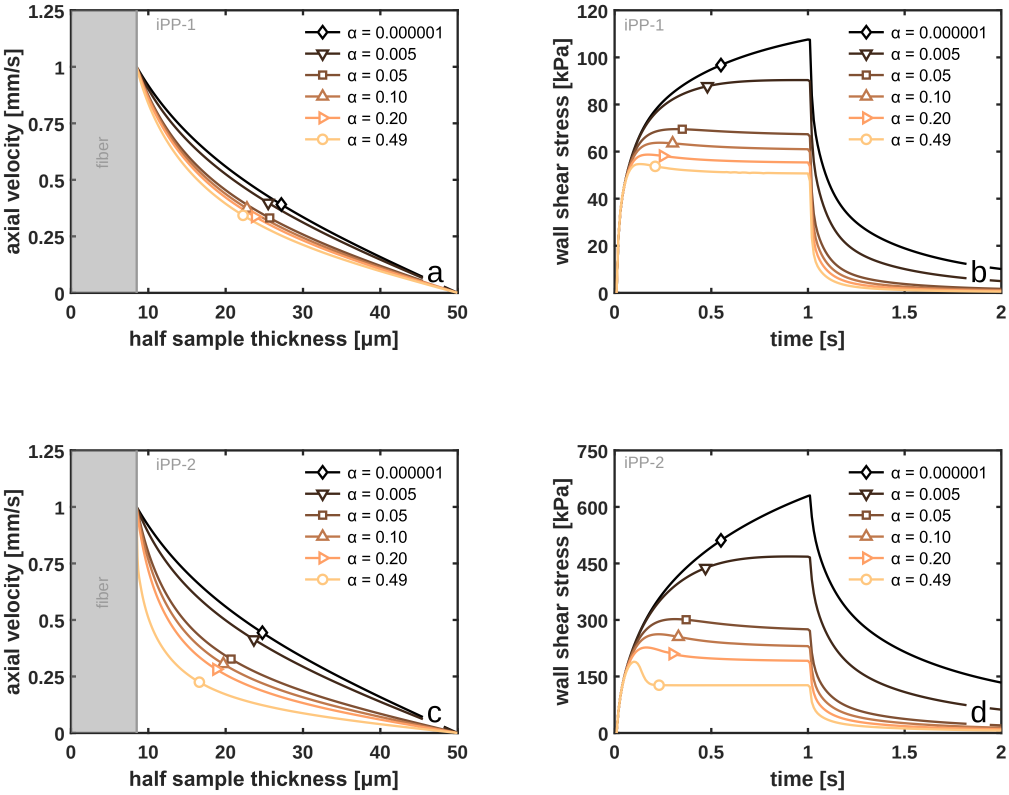

Figure S3: The steady-state velocity profiles over the sample height after application of a step-velocity profile to the fiber surface of 1 mm/s during 1 s for a) iPP-1 and c) iPP-2. The respective wall shear stresses are given in b) for iPP-1 and d) for iPP-2.

Effect of the applied velocity profile

In the remainder of this additional information, the effect of inconsistencies in the experimentally applied velocity profile on the deformation and stress state of the material is discussed further. The main text of this

article (Figure 10) highlights the relative small increase in shear stress when the maximum velocity achieved during pulling is 2.5 times higher compared to the prescribed step-velocity profile. In addition the effect of sample thickness on that shear stress is discussed (Figure 11), which demonstrates that unless confined crystallization is considered, i.e. the sample height is in the order of the fiber thickness, the steady-state wall shear stress changes only marginally with reducing the sample thickness. In Figure S4 the applied velocity profile along with the predicted wall shear stress over time is presented for the situation that the maximum fiber velocity is equal to 1 mm/s, and the total shear time is equal to 1 s. In that case the total displacement of the fiber is not equal to 1 mm, yet the influence thereof on the wall shear stress is negligible. A similar observation can be made when we consider a situation in which the total displacement is always 1 mm, and the maximum attained velocity is always 1 mm/s; any start-up phenomenon would then lead to an increase in total shear time, as shown in Figure S5. Combining these results with the observations described in the main article, we can solely come to the conclusion that within reasonable limits inconsistencies in experimental conditions will not lead to a significant difference in shear induced nucleation rate, and that the molecular characteristics of the material under study is the determining factor here.

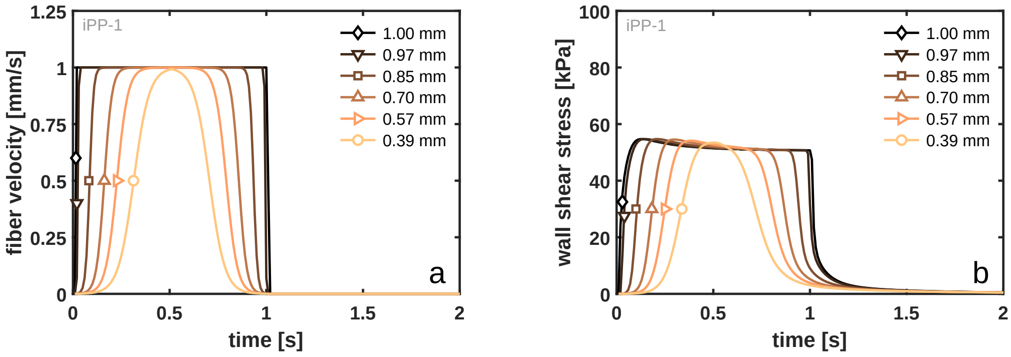

Figure S4: a) The prescribed velocity profiles at the fiber surface when the maximum velocity and shear time are kept constant. b) the corresponding wall shear stress at the fiber surface as a function of time. These simulation results represent the iPP-1 matrix material.

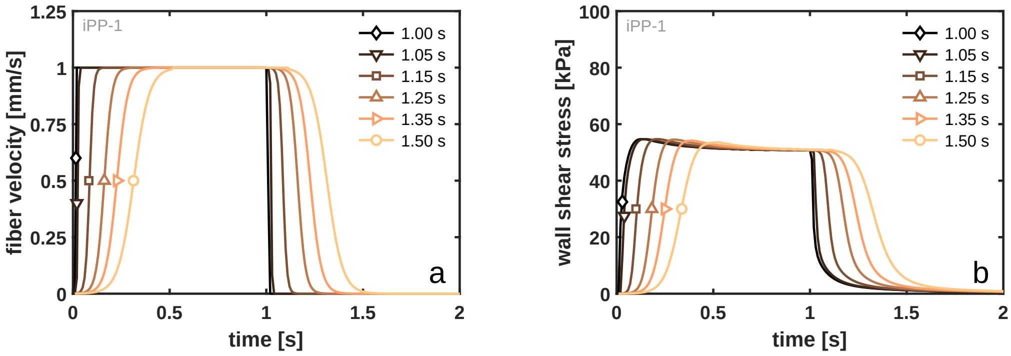

Figure S5: a) The prescribed velocity profiles at the fiber surface when the maximum velocity and total displacement are kept constant. b) the corresponding wall shear stress at the fiber surface as a function of time. These simulation results represent the iPP-1 matrix material.
